# Barriers Against and Motivations for Quitting Smoking during the COVID-19 Health Crisis: Results of a Qualitative Study in France

**DOI:** 10.3390/ijerph192013051

**Published:** 2022-10-11

**Authors:** Romain Guignard, Guillemette Quatremère, Anne Pasquereau, Cécile Jartoux, Laure Salvaing, Guillaume Caline, François Beck, Viêt Nguyen Thanh

**Affiliations:** 1 Santé Publique France, The French National Public Health Agency, 12 Rue Du Val d’Osne, CEDEX, 94415 Saint-Maurice, France; 2 Kantar Public, 3 Avenue Pierre Masse, 75014 Paris, France

**Keywords:** COVID-19, tobacco, smoking cessation, motivations, barriers, qualitative study

## Abstract

The COVID-19 epidemic and its psychological, economic and social consequences could have an impact on the evolution of tobacco-smoking prevalence and attitudes towards quitting. The aim of this study is to analyse the specific characteristics of the motivations for and barriers against smoking cessation in this period. The study is based on qualitative data collected from late 2020 to early 2021 in France from 89 smokers with a low or intermediate socio-economic level. Among the motivations for quitting smoking, health concerns and the financial cost of cigarettes carried increased importance among the smokers in this period. Inversely, perceived stress, isolation, and a lack of social or healthcare support prevented some smokers from attempting to quit. These results are useful for explaining the evolution of smoking prevalence and preparing future interventions in the context of the health crisis and its aftermath. They highlight the relevance of educational messages, of the promotion of validated smoking-cessation aids and, in particular, remote support, as well as the implementation of community-based actions.

## 1. Introduction

Tobacco consumption is one of the main preventable risk factors for premature deaths (i.e., 7.7 million in 2019) and diseases worldwide [1]. In France, as in most Western countries, smoking prevalence has decreased in recent years, while remaining at a high level. However, 2020 saw a break from this trend, as the prevalence of daily smoking stabilised in the first half at 25.5% of the adult population and even increased among the lowest socio-economic categories [2]. In addition, for the past few years, social inequalities linked to smoking have remained very marked, with higher prevalence among people with low incomes or the unemployed, for example [3,4,5].

The years 2020 and 2021 were marked by the COVID-19 health crisis and its psychological, economic and social consequences [6]. In France, two national lockdowns were implemented, in spring and late 2020, followed by a national curfew until mid-2021. Over the same period, local curfews also supplemented national measures. These exceptional health and regulatory contexts significantly affected personal and professional lives and could have had an impact on smoking habits. In addition, as situations triggering psychological tensions are associated with an increase in addictive behaviour, the monitoring of changes in smoking habits during the health crisis was particularly important [7,8].

In France, variations in tobacco use were observed during the first population-wide lockdown in spring 2020, in terms of both upward and downward trends, following changes in work situation, housing conditions and, more broadly, quality of life, as well as the usual reasons (such as stress management and sociability) [9,10]. The proportion of smokers who increased their tobacco consumption was nevertheless higher than that of those who decreased it, and certain risk profiles were identified, such as being a woman, having anxiety or depression, having a higher level of education, being unemployed or working from home [10].

The reasons for quitting smoking reported by smokers outside the pandemic period are diverse. In France, in 2010, the main reasons mentioned by smokers and ex-smokers for smoking cessation were health preservation (for 55% of smokers and 75% of ex-smokers), relationships with friends and family, including awareness of the risks of passive smoking and requests by relatives (for 40% of smokers and 45% of ex-smokers) and the price of tobacco (for 27% of smokers and 25% of ex-smokers) [11]. A qualitative study conducted in Poland also revealed health concerns, the cost of cigarettes, a ban on smoking at home or at work, odour, pregnancy and breastfeeding [12]. Among the reasons for relapse, the same study mentioned stress and the need to reduce it by smoking, the need for some smokers to rediscover the pleasure of cigarettes, and being surrounded by smokers at home or at work [12]. Furthermore, a review of the literature reported common barriers to smoking cessation among vulnerable populations: the use of tobacco as a stress-management tool or the high prevalence and acceptability of tobacco use in these groups, as well as the lack of support from healthcare professionals to quit smoking and difficulties in accessing resources for these disadvantaged populations [13].

Several studies have also been carried out on the motivations for and barriers against smoking cessation during COVID-19, concerning, for example, risk perception or the constraints imposed by lockdown measures. However, these studies were conducted on specific populations of smokers and in the context of clinical trials, ranging from evaluations of smoking-cessation interventions for smokers who wished to quit smoking [14,15] to lung-cancer-screening programmes for elderly subjects [16]. At the beginning of the epidemic, the hypothesis of a protective effect of nicotine on SARS-Cov2 infection was suggested as a reason for discouraging smoking cessation, although the causal relationship was not conclusively demonstrated [17].

It cannot be ruled out that the COVID-19 epidemic interrupted the decline in smoking prevalence observed in France for several years. The study of the barriers against and motivations for quitting smoking in the general population is useful in explaining the change in the trend and for proposing interventions in this context. The aim of this study is to analyse the specific characteristics of the motivations for and barriers against quitting smoking in times of health crisis, among smokers with a low or intermediate socio-economic level, based on qualitative data collected from late 2020 to early 2021 in France.

## 2. Materials and Methods

### 2.1. Participants

The participants in this study were interviewed as part of two qualitative post-tests conducted among smokers with an intermediate or low socio-economic level. The post-tests aimed to evaluate two different French communication campaigns for smoking cessation in terms of visibility, perceptions, perceived effects on change behavior, etc. The first post-test was carried out between November and December 2020, and the second in February 2021. The participants were recruited mainly by the Kantar Public Institute from a database of individuals who agreed to participate in market research, completed for the first post-test by participants registered to the “Mois sans tabac” campaign run by Santé publique France [18]. The inclusion criteria were as follows: being 18 to 65 years of age, being a smoker, considering quitting smoking in the coming months, having a level of education less than or equal to 2 years after the completion of the baccalaureate, being from an intermediate socio-professional category (farmer, artisan, shopkeeper, middle executive, teacher, technician…) or lower (routine or manual occupation), having a computer with an internet connection, and having not participated in a study for Kantar Public in the last 6 months. For the post-test carried out at the end of 2020, a specific sample of smokers in a precarious financial or professional situation (unemployed, beneficiary of minimum allowances, employed in manual work or on a temporary/fixed-term contract with an income close to minimum wage) was interviewed, making it possible to study specific characteristics of this target. Diversity was sought in terms of gender, region, type of conglomeration (urban, suburban, rural), and having children.

### 2.2. Data Collection

For each time period (November to December 2020, February 2021), the data were collected during focus groups of 4 to 5 people lasting 2 h to 2 h 30 min, and one-hour individual interviews conducted via videoconferencing due to the need to limit social interaction during this period. The individual interviews during the post-test at the end of 2020 were conducted with smokers in a precarious financial or professional situation only. The focus groups and interviews were conducted by psychosociologists and Kantar Public study managers, trained in facilitation methods, using semi-structured interview grids based on an inductive approach [19]. The main objective of the two post-tests was to study perceptions of recently disseminated tobacco-prevention campaigns. However, questions specific to the present study, i.e., about smokers’ attitudes towards smoking cessation during the health crisis, were introduced at the start of the interview (perceived benefits and disadvantages of quitting, previous attempts to quit, intention to quit, fears, envisaged means, impact of the epidemic and the resulting restrictions on tobacco consumption, motivation and perceived ability to quit). The interviews were recorded with the written consent of participants before being transcribed and anonymized. Participants were paid EUR 45 for focus-group participation and EUR 25 for individual interviews. The present study adheres to the conventions of the National Data Protection Authority (Commission Nationale Informatique et Liberté (CNIL)). Written informed consent was obtained from participants before participation.

### 2.3. Data Analysis

The transcriptions were read by two Kantar Public study officers, and compared with the initial analyses of the psychosociologists responsible for data collection. Analysis categories were discussed and identified, summarising the main points of the interview grids. The analysis of the content produced was then read and discussed by the authors. Thematic saturation of the collected data was reached in this sample.

## 3. Results

### 3.1. Description of the Sample and Consumption Habits during the COVID-19 Epidemic

In total, 89 smokers were interviewed between November and December 2020 (*n* = 40) and in February 2021 (*n* = 49). Sixty-six smokers were interviewed in the focus groups and twenty-three in individual interviews, including eight interviews with smokers who were in a precarious financial or employment-related situation at the end of 2020. The sociodemographic characteristics of the respondents are presented in Table 1. All the individuals were from low or intermediate socioprofessional categories.

The COVID-19 health crisis led to various changes in tobacco consumption among the respondents. Among the participants reporting an increase in their consumption, who comprised the majority of this sample, the reasons given were stress or boredom, which could in part be linked to changes in employment status (remote working or partial unemployment). Individuals claiming to have smoked less as a result of the health crisis mentioned limited social opportunities (at work or when going out) as reducing their consumption, which had previously mainly occurred in sociable contexts. These participants were more frequently light or occasional smokers. Finally, some of the participants did not change their level of tobacco use, due to their unchanged employment situation or the replacement of social cigarettes with cigarettes intended to relieve stress or boredom. Thus, the changes in consumption appeared to be closely linked to the work situations of the individuals during the epidemic and also to their relationship with cigarettes (social convention or stress management).

### 3.2. Motivations to Quit Smoking

The three reasons for smoking cessation mentioned by most of the smokers questioned in this study were health, the cost of tobacco, and the willingness to regain a form of freedom. These reasons were shared regardless of the participants’ profiles, but slight differences were observed.

Health was mentioned more by chronic smokers, who cited the immediate impact of stopping on the symptoms they attributed to tobacco and which affected their quality of life (shortness of breath, fatigue, cough, chronic bronchitis). The fear of long-term diseases, cancer, and even death, was mentioned less. In older people, the desire to age well, particularly in order to spend more time with their grandchildren, was also mentioned. Furthermore, the majority of the smokers surveyed felt that smoking could be responsible for worsening COVID-19 symptoms.

*“And the physical effect on health. We don’t feel it right away, but I feel it today. The congestion, the phlegm. When we’re younger, we are more lively. But when we get older…”*

(Individual interview, 50-year-old man)

The cost of smoking seemed to occupy an increasing role in the participants’ desire to quit smoking. Some of the smokers, especially those with children, had been feeling guilty about spending so much on smoking, considering that they could put the money to better use, and some smokers in precarious situations said they could no longer afford to smoke. This refers in particular to the significant increases in tobacco prices recorded in recent years, and the crisis sometimes increased the contribution of this economic factor to the desire to quit, particularly for smokers in difficult financial situations.

*“This summer, I said to myself, ‘You lost your job because of the crisis’, and that the savings made are the most important. Spending 250 euros on ciggies is a large amount going up in smoke, and as there were two of us in the same situation, I said ‘We have to stop’.”*

(Individual interview, 26-year-old woman)

The weariness at the idea that smoking takes over daily life, often at the expense of the amount of quality time spent with friends and family, was mentioned as a motivation for quitting.

*“I had noticed how much my smoking was taking over everything. At 7 a.m. I already knew that at 9 a.m. I was going to have a coffee and smoke a cigarette. It’s frightening.”*

(Individual interview, 50-year-old man)

Among other reasons for stopping, the inconveniences associated with smoking for loved ones, such as odour, was frequently reported.

*“The smell, it doesn’t smell very good. Not polluting others. Respect for others. Bad example for children.”*

(Focus group, 40–64 years)

Other reasons were mentioned by the respondents, but less systematically: setting a good example for their children or grandchildren, family pressure, especially that of children, awareness of passive smoking for which the participants were responsible, the potential for more free time, or events such as pregnancy.

### 3.3. Barriers to Smoking Cessation

Among the smokers interviewed in this study, fairly similar barriers to smoking cessation were mentioned.

Above all, the fear of the symptoms or inconveniences associated with withdrawal, both physical and psychological, was described. For example, weight gain and irritability were cited particularly frequently as having been observed in family members or even during previous attempts to quit. The relative importance given to these two symptoms appeared to depend on the gender of the interviewee, since the women interviewed seemed to be more affected by weight gain, while men were more concerned about an increase in their nervousness.

*“The fear of lack, fear of feeling on edge. What we see in the films is also reality.”*

(Individual interview, 43-year-old man)

*“Weight gain, nervousness; you’re not patient when you smoke. When I stopped, I was always thinking about it: drinking coffee, during breaks. When you do these things in the days after, you say to yourself, oh no, I don’t smoke anymore. It’s the brain that reminds you before the rest.”*

(Focus group, 30–40 years)

Stopping smoking can also imply separation from pleasure and from forms of support in daily life, which could have been especially complicated for those most affected by the crisis.

*“We know all that [the risks associated with smoking] but you must want to stop in fact because cigarettes remain our sole pleasure.”*

(Individual interview, 36-year-old man)

*“It’s true that a cigarette, when you’re on your own, is a form of company.”*

(Individual interview, 47-year-old man)

*“It’s my emotions, I can’t escape them, smoking a ciggie helps me deal with my emotions, which is why I do it again.”*

(Individual interview, 26-year-old woman)

For the majority of the respondents, particularly those whose consumption increased or plateaued during the first lockdown of 2020, the context of a prolonged health and economic crisis combined with an overabundance of information messages, particularly health information, made the desire to stop even more difficult due to the stress that prompted them to consume cigarettes.

*“It’s a time of stress and personally stress makes me smoke more, I find comfort in smoking a cigarette.”*

(Individual interview, 33-year-old woman)

*“We’re going through things we’ve never known and there’s quite a lot of negativity around us, about what we’re living. It’s so dark that it doesn’t encourage stopping smoking.”*

(Focus group, 40–64 years)

Anticipating the breaking of social bonds that had been created around smoking could also discourage quitting. When cigarettes are associated with times spent with friends or co-workers, the fear of no longer belonging to the group, of missing out on cigarette-break conversations, may be present.

*“Yes, there are good times with people [colleagues] too, like the after-lunch ciggie”*

(Focus group, 18–34 years old)

In some heavy smokers, the social dimension is so important that quitting can herald a form of identity crisis. In our study, this was the case among those who considered their status as smokers to be truly integral to their personalities, especially if they had smoked since a young age.

*“When I started smoking again after having stopped for twenty years, I finally felt like myself again!”*

(Focus group, 50–65 years)

Difficulties quitting smoking are closely related to how withdrawal is perceived. Thus, most of the participants interviewed considered quitting smoking as an individual act based on their willpower alone. The importance of support can be completely omitted when faced with the perception of willpower as the determining factor in quitting successfully. This resigned and solitary viewpoint can create a fear of failure, which is likely to encourage individuals to abandon any commitment they may have made to stopping.

*“I would like to stop like I did the first time, through willpower. Without anything else. If I can.” “I don’t want to take substitutes, chewing gums, it didn’t help me. I would like to try with willpower alone.”*

(Focus group, 40–64 years)

In fact, a lack of support within close circles could discourage some smokers who wish to quit, and the presence of smokers in close circles could make the withdrawal process more difficult.

*“I’m really all alone. I don’t see who I could turn to for help, you know, the family, it’s complicated.”*

(Individual interview, 54-year-old woman)

Quitting smoking can also be difficult due to a real lack of awareness of existing tools or support programmes. The idea of help from a tobacco specialist was, for example, unknown to the majority of the interviewees.

*“I’ve tried [to quit smoking] really briefly, but I feel like I can’t do it without help, I don’t know where to start. I would like to, but I don’t have a clue where to start.”*

(Individual interview, 36-year-old woman)

Lastly, a greater suspicion towards public authorities and a greater degree of lassitude around health messages was observed among the participants from the lowest-income social categories. These attitudes provoked less acceptance of public-health messages and, therefore, the rejection of campaigns that encouraged quitting, which continued to circulate during the crisis period.

*“I don’t watch the news anymore. I’m fed up with COVID, I can’t take anymore.”*

(Focus group, 40–64 years)

## 4. Discussion

### 4.1. Summary of Results, Interpretation and Comparison with the Literature

In this qualitative study conducted on smokers in low or intermediate socio-economic categories who wanted to quit smoking in the coming months, a large part of the motivations for and barriers against smoking cessation were not specific to the COVID-19-epidemic period.

The main reasons for quitting smoking were health, the cost of tobacco, and the desire to regain a form of freedom. These three factors correspond with the usual motivations observed outside the pandemic period [12,20]. They were cited without any particular distinction according to gender, but differences were observed according to age—health was of greater concern to the elderly in the sample—and standard of living. Those with dependent children and the least financially comfortable more often highlighted the cost of tobacco as a motivation. Specific health events, such as pregnancy and the influence of family members in the broad sense of the term (peer pressure or pressure from children, risks related to passive smoking) were also cited based on individual circumstances.

Most of the barriers to smoking cessation described by the respondents were also identified in other studies outside the pandemic context, such as fear of symptoms or discomforts related to withdrawal, fear of failure or a feeling of loss (of pleasure, a way to fill boredom or loneliness, social ties, identity in a way) [12,13,21,22]. Gender differences were observed; the fear of weight gain was more often noted by women, and the fear of being on edge was more often noted by men. However, the use of cigarettes as a tool for managing stress or emotions, which is believed to be more frequent in women [23,24], was not found in this study.

The lack of support and lack of knowledge about the help available are two other barriers that appear to be important according to the smokers interviewed: some referred directly to the lack of support in their close circle, and there was also a strong lack of awareness about support programmes or healthcare professionals specialising in smoking-cessation support. Conversely, individual will was strongly highlighted and valued by some of the respondents. This can undoubtedly be explained in part by the lack of knowledge on the help available and, perhaps, for some smokers, by a difficulty in admitting that they may need help, particularly in the form of medication, which is usually reserved for the sick. In a recent study conducted in the United States, Australia, Canada and England, almost 40% of smokers reported that they did not ask for any help during their last attempt to quit [25]. Even lower levels of use of smoking cessation aids were observed in Europe [26] and France [27].

Nevertheless, some factors were amplified by the COVID-19 crisis. Thus, although health concerns appeared before the pandemic as a motivation to quit smoking, the perception that smoking could be responsible for aggravating COVID-19 symptoms, as described in the scientific literature [28,29,30,31], seems to have reinforced the importance given to this criterion in the pandemic situation. These results are confirmed by other qualitative and quantitative studies [15,16]. For example, the perception of the increased risk of COVID-19 among smokers is associated with an increased motivation to quit smoking [32]. In a study of nearly 7000 Australian, Canadian, British and American smokers, nearly half reported thinking about quitting smoking due to the pandemic [33]. Although some of the respondents in our study reported having heard a conflicting message in spring 2020, namely that nicotine was a protective factor against COVID-19 [34], the majority of smokers interviewed qualified these studies as “urban legends” following numerous cautionary messages from scientific authorities [35,36]. Finally, certain profiles were likely to be more sensitive than others to the health factor: presenting risk factors or comorbidities likely to aggravate the COVID-19 disease would encourage individuals to reduce their consumption or attempt to quit smoking [37,38].

As in other studies, the cost of tobacco was also a strong motivation to quit smoking [16]. However, smokers on the lowest incomes have been heavily affected by the continued increase in tobacco prices in France since 2017—with tax increases applied in March and November 2020 raising the price of a pack of cigarettes to EUR 10 at the end of 2020—combined with an economic situation often made more precarious by the health crisis. In May 2020, a quarter of French people saw a deterioration in their financial condition [39]. In a study conducted in Canada, Australia, the United Kingdom and the United States, having a precarious financial situation was associated with an increase in the willingness to quit smoking in response to the COVID-19 epidemic [33].

Other barriers identified in our study could have been generated or strengthened by the crisis situation. Thus, the isolation of some of the smokers interviewed, amplified by lockdowns and curfews, could have been a factor in their discouragement, which would be in line with the results of a study establishing a relationship between increased consumption and living alone during the pandemic [38], or even a decrease in motivation for smoking cessation due to boredom and a lack of social activities [14,15].

Finally, the stress produced by the crisis seems to have been a limiting factor in the desire and attempt to quit smoking. First, anxiety and depression could have increased tobacco use, as has been observed in France [9] and abroad [40,41,42]. Second, this state of mental health could have made the smokers less able to stop [43]: a reduction in perceived self-efficacy is noted in several studies [14,15,16]. However, according to a Dutch study, the relationship between the stress caused by the health crisis and changes in smoking habits is ambivalent: it concluded that stress was systematically associated with changes in consumption, but that this shift could take the form of increase or reduction. The stress caused by COVID-19 has prompted some smokers to pay more attention to their health, as described above, while others have offset the stress generated by boredom and restricted mobility by increasing their consumption [44].

### 4.2. Strengths and Limitations

The present study may have been subject to reporting bias, including social-desirability bias in the case of the group surveys or those interviewed in the presence of investigators. Moreover, studies conducted on the Internet or via videoconferencing may be subject to selection bias, in that they require the people recruited to own a computer or smartphone with an internet connection and be relatively comfortable using these tools. Furthermore, the data used in this study were taken from post-tests, the main objective of which was not to explore the barriers against and motivations for quitting smoking in depth. This was a secondary analysis that sought to identify possible changes in motivation and the appearance of new barriers.

Our study also has strengths. First of all, there are studies on the change in tobacco use during the crisis and the reasons for these changes but, to our knowledge, there are no studies that have sought to describe the motivations for and barriers against smoking cessation during the COVID-19 epidemic in the general population. In addition, our qualitative method has several advantages for exploratory research on the perceptions of smokers: the in-depth, semi-directed group interviews helped the participants to discuss concerns and motivations in the discussions, which is more difficult to achieve through questionnaires composed mainly of closed questions. In addition, the samples created made it possible to combine two perspectives of interest. Firstly, the study involved smokers from diverse categories in terms of age, gender, geographical location and professional situation, which reinforces the external validity of our conclusions. Secondly, the sample offered a focus on socioeconomically disadvantaged smokers, a population that remains a priority for prevention actions in France and in most high-income countries, given the significant social inequalities associated with tobacco use.

## 5. Conclusions

This study reviews the motivations that should be supported and the additional barriers to overcome with regard to smoking cessation in the context of health crises. Thus, vulnerability to disease can be an opportune driver at such a time, as knowledge of the effects of smoking on the consequences of infection can trigger certain smokers to give up tobacco, particularly if they have fragile health [37,45]. However, this argument must be handled with great care insofar as it could generate tension for those who may perceive these messages as the exploitation of this relationship by the public authorities. In our sample, this finding seems to have been stronger among the most socioeconomically vulnerable populations. Therefore, information on these risks should come with a compassionate, non-guilt-inducing message that presents tangible solutions [46], in order to avoid rejection reactions and to support smokers with fragile mental health.

The cost of tobacco is a growing motivation, especially for people in difficult financial situations that could be aggravated by the crisis. It is necessary to support these socioeconomically vulnerable populations, especially since they have less access to help, but need it more (greater physical dependence on nicotine, more smokers among relatives, friends and colleagues, etc.).

The recovery from the epidemic crisis could be conducive to tobacco-prevention campaigns, with more audiences being more receptive to health issues, if these campaigns are able to emerge from among the swathe of health messages currently issued. More generally, in times of crisis and beyond, it is important to continue to educate smokers about smoking cessation, make help accessible, promote validated aids (medical and psychological) and increase awareness about effective remote-support tools. The implementation of community-based actions promoting smoking cessation, which have been particularly jeopardised during the epidemic, could also be a path to renewed action.

## Figures and Tables

**Table 1:** Sociodemographic description of smokers interviewed during the qualitative post-tests (n), November 2020–February 2021.

	Focus Groups	Individual Interviews	Total
Gender			
Male	31	11	42
Female	35	12	47
Age			
18–34 years	28	9	37
35–49 years	24	9	33
50–65 years	14	5	19
Type of conglomeration			
Rural area	7	5	12
Suburban area	23	5	28
Urban area	36	13	49
Total	66	23	89

## Data Availability

The data presented in this study are available on request from the corresponding author.
